# Identification of an Alternative Glycyrrhizin Metabolite Causing Liquorice-Induced Pseudohyperaldosteronism and the Development of ELISA System to Detect the Predictive Biomarker

**DOI:** 10.3389/fphar.2021.688508

**Published:** 2021-05-17

**Authors:** Kan'ichiro Ishiuchi, Osamu Morinaga, Tetsuhiro Yoshino, Miaki Mitamura, Asuka Hirasawa, Yasuhito Maki, Yuuna Tashita, Tsubasa Kondo, Kakuyou Ogawa, Fangyi Lian, Keiko Ogawa-Ochiai, Kiyoshi Minamizawa, Takao Namiki, Masaru Mimura, Kenji Watanabe, Toshiaki Makino

**Affiliations:** ^1^ Department of Pharmacognosy, Graduate School of Pharmaceutical Sciences, Nagoya City University, Nagoya, Japan; ^2^ Department of Natural Medicines, Daiichi University of Pharmacy, Fukuoka, Japan; ^3^ Center for Kampo Medicine, Keio University School of Medicine, Tokyo, Japan; ^4^ Department of Otorhinolaryngology and Head and Neck Surgery, Clinic of Japanese Oriental (Kampo) Medicine, Kanazawa University Hospital, Kanazawa, Japan; ^5^ Department of Oriental Medicine, Kameda Medical Center, Kamogawa, Japan; ^6^ Department of Japanese Oriental (Kampo) Medicine, Graduate School of Medicine, Chiba University, Chuo-ku, Japan

**Keywords:** kampo medicine, side effect, liquorice, glycyrrhizin, pseudoaldosteronism, sex differences

## Abstract

Liquorice is usually used as crude drug in traditional Japanese Kampo medicine and traditional Chinese medicine. Liquorice-containing glycyrrhizin (GL) can cause pseudohyperaldosteronism as a side effect. Previously, we identified 18*β*-glycyrrhetyl-3-*O*-sulfate (**3**) as a GL metabolite in Eisai hyperbilirubinuria rats (EHBRs) with the dysfunction of multidrug resistance-related protein (Mrp2). We speculated that **3** was associated with the onset of liquorice-induced pseudohyperaldosteronism, because it was mainly detected in serum of patients with suspected to have this condition. However, it is predicted that other metabolites might exist in the urine of EHBRs orally treated with glycyrrhetinic acid (GA). We explored other metabolites in the urine of EHBRs, and investigated the pharmacokinetic profiles of the new metabolite in EHBRs and normal Sprague-Dawley rats. We further analyzed the serum concentrations of the new metabolite in the patients of pseudohyperaldosteronism. Finally, we developed the analyzing method of these metabolites as a preventive biomarker for the onset of pseudohyperaldosteronism using an enzyme-linked immunosorbent assay (ELISA). We isolated a new GL metabolite, 18*β*-glycyrrhetyl-3-*O*-sulfate-30-*O*-glucuronide (**4**). Compound **4** significantly inhibited rat type-2 11*β*-hydroxysteroid dehydrogenase (11*β*-HSD2) and was a substrate of both organic anion transporter (OAT) 1 and OAT3. Compound **4** was also detected in the serum of patients with suspected pseudohyperaldosteronism at an approximately 10-fold lower concentrations than **3**, and these concentrations were positively correlated. Compound **4** showed a lower serum concentration and weaker inhibitory titer on 11*β*-HSD2 than **3**. We developed an enzyme-linked immunosorbent assay system using an anti-18*β*-glycyrrhetyl-3-*O*-glucuronide (3MGA) monoclonal antibody to measure the serum concentration of **3** to facilitate the measurement of biomarkers to predict the onset of pseudohyperaldosteronism. Although we found **4** as the secondary candidate causative agent, **3** could be the main potent preventive biomarker of liquorice-induced pseudohyperaldosteronism. Compound **3** was detected in serum at a higher concentration than GA and **4**, implying that **3** may be a pharmacologically active ingredient mediating not only the development of pseudohyperaldosteronism but anti-inflammatory effects in humans administered GL or other liquorice-containing preparations.

## Introduction

**FIGURE 1:**
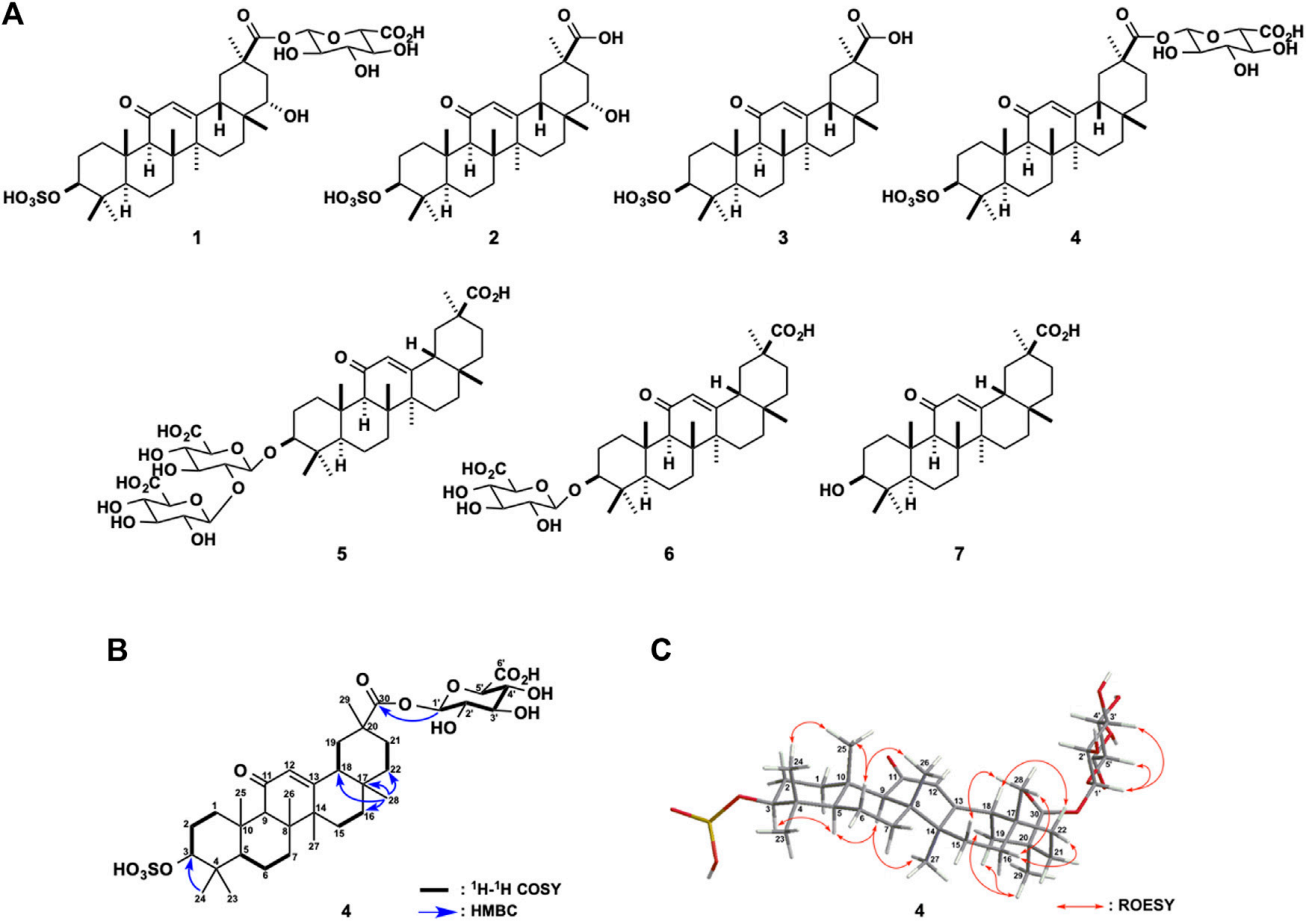
**(A)** Chemical structures of glycyrrhizin (GL, **5**) and its metabolites (**1**−**4**), 18*β*-glycyrrhetyl-3-*O*-glucuronide (3MGA, **6**), and glycyrrhetinic acid (GA, **7**) **(B)** Key two-dimensional (2D) nuclear magnetic resonance (NMR) correlations for **4** **(C)** Selected rotating frame overhause effect spectroscopy (ROESY) correlations and relative stereochemistry of **4**.

Liquorice, the root or stolon of *Glycyrrhiza uralensis* or *G. glabra*, is a natural material commonly used as a sweetener for foods as well as a crude drug used in traditional Japanese Kampo medicine and traditional Chinese medicine (TCM). Its component glycyrrhizin (**5**) (GL; Figure 1A) has various pharmacological actions such as anti-allergy, anti-inflammation, anti-ulcer, anti-virus, anti-androgenic, and hepatorptective effects (Isbrucker and Burdock, 2006). Pseudohyperaldosteronism, a well-known side effect of liquorice, is characterized by hypertension, oedema, and hypokalaemia (Conn et al., 1968; Stewart et al., 1987). The onset of pseudohyperaldosteronism is associated with the inhibition of type-2 11*β*-hydroxysteroid dehydrogenase (11*β*-HSD2) in renal distal tubular epithelial cells by some GL metabolites (Ploeger et al., 2001). Under normal conditions, cortisol, which has a similar affinity to the mineralocorticoid receptor as aldosterone, is converted by 11*β*-HSD2 to cortisone, which has a low affinity for the receptor. When 11*β*-HSD2 is inhibited by GL metabolites, the local cortisol concentration increases, which stimulates the mineralocorticoid receptor. Consequently, sodium reabsorption and potassium excretion are accelerated, resulting in symptoms such as hypokalaemia, hypertension, and oedema (Ploeger et al., 2001).

Recently, we found 22*α*-hydroxy-18*β*-glycyrrhetyl-3-*O*-sulfate-30-glucuronide (**1**), 22*α*-hydroxy-18*β*-glycyrrhetyl-3-*O*-sulfate (**2**), and 18*β*-glycyrrhetyl-3-*O*-sulfate (**3**; Figure 1A) as GL metabolites in the urine of Eisai hyperbilirubinuria rats (EHBRs) orally treated with glycyrrhetinic acid (**7**, GA; Figure 1A) (Morinaga et al., 2018; Ishiuchi et al., 2019). Orally administered GL is hydrolysed by intestinal bacteria to GA and appears in circulating blood (Akao et al., 1994). GA then translocates to the liver where it is metabolised to compounds **1**–**3**, and 18*β*-glycyrrhetyl-3-*O*-glucuronide (**6**, 3MGA; Figure 1A) by sulfotransferase (SULT) 2A1 (Takahashi et al., 2019), some cytochrome P450 (CYP) enzymes, and some glucuronyltransferases (Kato et al., 1995; Makino et al., 2008; Makino et al., 2012; Morinaga et al., 2018; Ishiuchi et al., 2019).

Previous studies suggested GA or 3MGA as causative agents of pseudohyperaldosteronism because they had a greater inhibitory effect on the activity of 11*β*-HSD2 than GL, which was not detected in the blood (Monder et al., 1989; Kato et al., 1995). Most of the GA, 3MGA, and compounds **1–3** are bound to albumin in the blood and cannot translocate to cells by simple diffusion. To inhibit 11*β*-HSD2, it has to be transported by some transporters into the renal tubular cells where it is actively expressed. Compounds **1–3** and 3MGA but not GA are substrates for organic anion transporter (OAT) 1 and OAT3 expressed on the vascular side of renal tubular epithelial cells. Since compounds **1–3** and 3MGA exhibit sufficient inhibitory effects on 11*β*-HSD2, we reported that all these compounds could cause pseudohyperaldosteronism (Makino et al., 2008; Makino et al., 2012; Morinaga et al., 2018; Ishiuchi et al., 2019).

Furthermore, we evaluated the association of the concentration of each GL metabolite in serum samples of patients with suspected pseudohyperaldosteronism with the laboratory data and various symptoms. Compound **3** but not **1**, **2**, and 3MGA were detected in the serum samples, which suggests the association of **3** with the onset of pseudohyperaldosteronism (Takahashi et al., 2019).

In this study, we isolated another new GL metabolite, 18*β*-glycyrrhetyl-3-*O*-sulfate-30-*O*-glucuronide (**4**), from the urine of EHBRs orally treated with GA and elucidated its structure based on spectroscopic data. We demonstrated the inhibitory effect of **4** on 11*β*-HSD2 and detected **4** in serum samples from patients with suspected pseudohyperaldosteronism. However, the concentration of **4** was lower than that of **3**, therefore, **3** was likely the major cause of pseudohyperaldosteronism. Then, we developed an enzyme-linked immunosorbent assay (ELISA) system to facilitate the measurement of the concentration of **3** in serum samples collected from the patients. In addition, we compared the differences in the pharmacokinetics of these GL metabolites between male and female EHBRs and humans.

## Material and Methods

### Chemicals, Reagents, Animals, and General Procedures

All chemicals, reagents, animals, and general procedures were the same as those described in our previous study (Morinaga et al., 2018; Ishiuchi et al., 2019). The animal experimental procedures were approved by the Animal Care Committee at the Graduate School of Pharmaceutical Sciences, Nagoya City University, Nagoya, in accordance with the guidelines of the Japanese Council on Animal Care.

### Isolation of Compound 4

EHBRs (6 week-old, Japan SLC, Hamamatsu, Japan) were reared and provided with drinking water containing 1 mg/ml GA suspended in 1% carboxymethylcellulose solution for 3 months. The urine of the rats was collected, pooled, filtered, and then 2.5 L of the urine was evaporated under reduced pressure (dried weight, approximately 40 g). The concentrated urine (1.4 L) was partitioned with ethyl acetate and 1-buthanol. The 1-buthanol-soluble part (25 g) was separated by ODS silica gel column chromatography with a mixture of methanol/H_2_O (1:4 → 1:0). A fraction eluted with methanol/H_2_O (3:2) was further separated by silica gel column chromatography (chloroform/methanol/H_2_O/trifluoroacetic acid 1:0:0:0 → 5:5:1:0.01), from which a fraction eluted with chloroform/methanol (1:1) was further purified using C_18_ high-performance liquid chromatography (HPLC, Cosmosil 5C_18_-ARII (Nakalai Tesque, Kyoto, Japan), 5 μm, 4.6 mm *i. d.* × 250 mm) with the following parameters: solvent, acetonitrile/H_2_O/trifluoroacetic acid (35:65:0.1); flow rate, 0.6 ml/min; detection 254 nm, to obtain compound **4** (3.2 mg).

Compound **4**: colorless amorphous solid [α]_D_^24^ + 107 (*c* 0.3, methanol); ultraviolet (UV) (methanol) *λ*_max_ 248 (*ε* 9782) nm; electronic circular dichroism (ECD) (methanol) *λ* (*Δε*) 230 (+8.3) nm; ^1^H-nuclear magnetic resonance (NMR) (deuterated methanol, 500 MHz) and ^13^C-NMR (deuterated methanol, 125 MHz), see Table 1; electrospray ionization tandem mass spectrometry (ESIMS) *m/z* 725 [M-H]^-^; high resolution (HR) ESIMS *m/z* 725.3212 [M-H]^-^ (calculated for C_36_H_53_O_13_S, 725.3207).

**TABLE 1:** ^1^H and ^13^C NMR Data (CD_3_OD) of compounds **4** and **1**.

	4			1	
Position	δ_H_ ^a^	δ_C_ ^b^	HMBC	δ_H_ ^a^	δ_C_ ^b^
1a	2.74 (1H, brd 13.5 Hz)	40.1	9, 25	2.74 (1H, d 13.5 Hz)	40.1
1b	1.05 (1H, nd^c^)			1.04 (1H, nd^c^)	
2a	2.07 (1H, brd 13.5 Hz)	25.2		2.08 (1H, brd 11.5 Hz)	25.2
2b	1.80 (1H, nd^c^)			1.78 (1H, nd^c^)	
3	3.96 (1H, dd 12.0, 4.0 Hz)	87.3	23, 24	3.96 (1H, m)	87.4
4		39.9	3, 23, 24		39.9
5	0.86 (1H, nd^c^)	56.6	1a, 7b, 23, 24, 25	0.86 (1H, nd^c^)	56.6
6a	1.64 (1H, brd 13.0 Hz)	18.6	5	1.65 (1H, brd 13.5 Hz)	18.6
6b	1.49 (1H, nd^c^)			1.49 (1H, nd^c^)	
7a	1.76 (1H, nd^c^)	33.8	6a, 26	1.76 (1H, nd^c^)	33.7
7b	1.45 (1H, nd^c^)			1.45 (1H, nd^c^)	
8		46.7	6a, 7a, 9, 15a, 26, 27		46.7
9	2.47 (1H, s)	63.0	7b, 8, 12, 25, 26	2.47 (1H, s)	63.1
10		38.2	1a, 5, 6a, 9, 25		38.1
11		202.6	9, 12		202.5
12	5.62 (1H, s)	129.1	18	5.61 (1H, s)	129.2
13		172.7	12, 15b, 18, 19b, 27		171.1
14		44.6	9, 12, 15a, 18, 26, 27		45.1
15a	1.88 (1H, nd^c^)	27.6	16a, 27	1.80 (1H, nd^c^)	27.2
15b	1.25 (1H, nd^c^)			1.29 (1H, m)	
16a	2.15 (1H, ddd 14.0, 14.0, 4.0 Hz)	27.4	15a, 18, 28	1.80 (1H, nd^c^)	20.3
16b	1.03 (1H, nd^c^)			1.48 (1H, nd^c^)	
17		32.9	15b, 16a, 18, 19a, 19b		38.4
			21a, 21b, 22b, 28		
18	2.26 (1H, dd 14.0, 3.5 Hz)	49.3	12, 16b, 19a, 19b, 22b	2.21 (1H, brd 13.5 Hz)	49.0
			28		
19a	1.92 (1H, nd^c^)	42.2	18, 21a, 29	1.89 (1H, brd 12.5 Hz)	41.6
19b	1.77 (1H, nd^c^)			1.80 (1H, nd^c^)	
20		45.2	19a, 19b, 21a, 22b, 29		45.1
21a	2.01 (1H, brd 9.0 Hz)	32.0	19a, 19b, 29	2.16 (1H, brd 11.0 Hz)	39.5
21b	1.45 (1H, nd^c^)			1.51 (1H, nd^c^)	
22a	1.45 (1H, nd^c^)	38.7	21b, 28	3.42 (1H, nd^c^)	76.1
22b	1.38 (1H, brd 10.5 Hz)				
23	1.06 (3H, s)	28.7	3, 24	1.07 (3H, s)	28.7
24	0.86 (3H, s)	16.9	3, 5, 23	0.86 (3H, s)	16.9
25	1.16 (3H, s)	17.0	9	1.16 (3H, s)	17.0
26	1.14 (3H, s)	19.3	7a, 7b, 9	1.14 (3H, s)	19.2
27	1.43 (3H, s)	23.8	15a, 15b	1.44 (3H, s)	23.9
28	0.82 (3H, s)	28.9	16a, 18, 22a	0.93 (3H, s)	25.5
29	1.21 (3H, s)	28.1	19b	1.25 (3H, s)	28.1
30		176.9	19b, 21b, 29, 1′		176.6
1′	5.53 (1H, d 8.0 Hz)	95.5	2′	5.53 (1H, d 7.5 Hz)	95.6
2′	3.40 (1H, dd 9.0, 8.0 Hz)	73.6	3′	3.42 (1H, nd^c^)	73.6
3′	3.45 (1H, dd 9.0, 9.0 Hz)	77.7	2′, 4′	3.45 (1H, nd^c^)	77.6
4′	3.59 (1H, dd 9.0, 9.0 Hz)	72.9	3′	3.60 (1H, dd 9.0, 9.0 Hz)	72.9
5′	3.90 (1H, d 9.0 Hz)	77.3	1′, 4′	3.90 (1H, d 9.0 Hz)	77.4
6′		171.9	4′, 5′		171.8

a 500 MHz.

b 125 MHz.

c nd: J-values were not determined because of overlapping with other signals.

### Determination of *in vitro* 11*β*-HSD2 Activity Using Rat Kidney Microsomes

Assays were conducted as described by Diederich et al. (2000) with the slight modifications as described in our previous report (Makino et al., 2012).

### Uptake of Compound 4 by Rat Kidney Slices and Cells Expressing OAT1 and 3

For the uptake study of **4** into rat kidney slices, we used the samples obtained in our previous study (Ishiuchi et al., 2019). For the uptake study of **4** into the cells transiently expressing OAT1 and OAT3, we used the same protocol as in our previous study (Makino et al., 2012). cDNAs encoding human OAT1 and OAT3 inserted into pGH19 were generously supplied by Prof. Mitsuru Sugawara (Hokkaido University, Sapporo, Japan), and then subcloned into a pCI-neo mammalian expression vector (Promega, Madison, WI, United States). We used the same pooled plasma of male EHBRs orally treated with GA for 12 h used in our previous study (Ishiuchi et al., 2019).

### Binding of Compound 4 to Albumin

We measured the binding ratios of **4** to albumin in pooled plasma of female EHBRs using the same protocol used in our previous study (Ishiuchi et al., 2019).

### Measurement of Compound 4 in Human Serum Samples

Human serum samples (100 µl) were pre-treated as described in the protocol reported in our previous study (Takahashi et al., 2019), and the concentration of **4** was measured using LC-tandem mass spectrometry (MS/MS) as described below. This study was conducted with the approval of the appropriate ethics committee at Keio University, Chiba University, Kanazawa University, and Kameda Medical Center.

### Evaluation of the Pathological Relationship Between Compound 4 and Pseudohyperaldosteronism Pathology

We recorded the following available clinical symptoms and laboratory data when blood samples were drawing from patients: age, sex, complications, concomitant medications, daily liquorice dose, duration of administration of liquorice-containing herbal preparations, symptoms (blood pressure and pedal oedema), and laboratory test values, namely: total protein, albumin, total and direct bilirubin, aspartate amino transferase, alanine amino transferase, blood urea nitrogen, creatinine, sodium, potassium, chloride, calcium, magnesium, prothrombin time, plasma renin activity or activated renin concentration, and plasma aldosterone concentration (blood test data), and urine concentrations of potassium and creatinine (urinalysis data). Samples were processed according to the individual institutional protocols, and all data were measured at each institution.

### Dot-Blot Analysis Using anti-3MGA-Monoclonal Antibody

An anti-3MGA-monoclonal antibody (mAb) and 3MGA-human serum albumin (HSA)-conjugate were developed using the same protocols established in our previous studies (Morinaga et al., 2018; Ishiuchi et al., 2019). GL, GA, 3MGA, compounds **1**–**4**, and bovine serum albumin (BSA, Sigma-Aldrich, St. Louis, MO, United States, 1 µg each) were spotted onto a Mustang E positively charged polyethersulfone membrane (Pall Co., East Hills, NY, United States), fixed, and stained with the anti-3MGA-mAb using the same method used in our previous study (Ishiuchi et al., 2019).

### ELISA System Using anti-3MGA-mAb

An ELISA system to measure the cross-reactivity of compounds **3** and **4** by using 3MGA-HSA-conjugate and anti-3MGA-mAb was used according to the protocol in our previous study (Ishiuchi et al., 2019). To measure the concentration of **3** in human serum samples using the system ELISA, **3** was dissolved in normal human serum (Sigma) to prepare standard solutions (16 nM–2.0 µM) and the samples or standard solutions (each 10 µl) and ethanol (40 µl) were well mixed and centrifuged (1.2 × 10^4^ × *g* for 7 min). The supernatant was dried up under nitrogen flow at 40°C overnight, and the residue was dissolved in Can Get Signal^®^ Solution 1 (Toyobo Co., Ltd: Osaka, Japan) (50 µl). The ELISA was conducted according to the protocol described in our previous study (Ishiuchi et al., 2019). When the observed values were more than 400 nM, the deproteinized serum samples were further diluted with Can Get Signal^®^ Solution 1 for two or four times and analyzed again to observe values from 15.6 to 400 nM.

### Pharmacokinetic Experiments of GA Metabolites in EHBRs Orally Treated With GA

Male and female EHBRs (9 week-old) were anaesthetized by an intraperitoneal injection of urethane (1 g/kg) and their jugular veins were exposed. GA suspended in 0.5% carboxymethylcellulose was then administered orally (200 mg/kg) to the unconscious rats, and blood samples were collected from the jugular vein while urine samples were collected using a metabolic cage at appropriate times over a 12 h period.

### LC-MS/MS Analysis for GA Metabolites

The concentrations of compounds **1**–**4**, 3MGA, GA, and GL in serum and urine samples were measured using an LC-MS/MS system (Quattro Premier XE; Waters, Milford, MS, United States) as described in our previous studies (Takahashi et al., 2019). Briefly, after diluting serum and urine samples with water to obtain suitable concentrations, they were digested with subtilisin (0.91 U/ml), followed by the addition of astragaloside IV (used as an internal standard; Fujifilm Wako Pure Chemicals, Osaka, Japan), and deproteinized using 80% ethanol. The supernatant was transferred into a vial and the concentrations of compounds **1**–**4**, 3MGA, GA, and GL in the samples prepared from plasma and urine were measured under the following conditions: column, Scherzo SM-C18 (3 μm, 3 mm *i. d.* × 150 mm; Imtakt, Kyoto, Japan); mobile phase (A) 5 mM ammonium acetate in H_2_O (B) 125 mM ammonium acetate in H_2_O/acetonitrile (1:4), at a flow rate of 0.3 ml/min, with the following gradient profile: A:B = 50:50–0:100 (0–2 min) and 0:100 (2–21 min). The transitions (precursor to daughter) monitored and retention times were as follows: ESI(+), 743.4 to 567.5 m*/z* (35 and 25 V for cone voltage and collision energy, respectively) for compound **1** (8.1 min); ESI(–), 565.5 to 96.5 m*/z* (80 and 60 V) for **2** (9.6 min); ESI(–), 549.5 to 96.5 m*/z* (80 and 60 V) for **3** (15.4 min); ESI(+), 727.3 to 551.2 m*/z* (30 and 25 V) for **4** (11.3 min); ESI(+), 647.6 to 453.6 m*/z* (40 and 20 V) for 3MGA (15.0 min); ESI(+), 471.3 to 91.0 m*/z* (40 and 20 V) for GA (13.4 min); ESI(+), 823.5 to 453.6 m*/z* (30 and 20 V) for GL (18.4 min); and ESI(+), 785.4 to 143.0 m*/z* (30 and 20 V) for astragaloside IV (3.0 min). Linear regressions over the concentration range of 2 nM to 2 µM for each compound were examined using the peak-area ratio of the compounds to their internal standards and the least-squares method (*r* ^*2*^ > 0.98). The detection limits for **1**, GL, and GA were 6.4 nM, and those for **2**, **3**, and **4,** and 3MGA were 3.2 nM.

### Statistics

**FIGURE 2:**
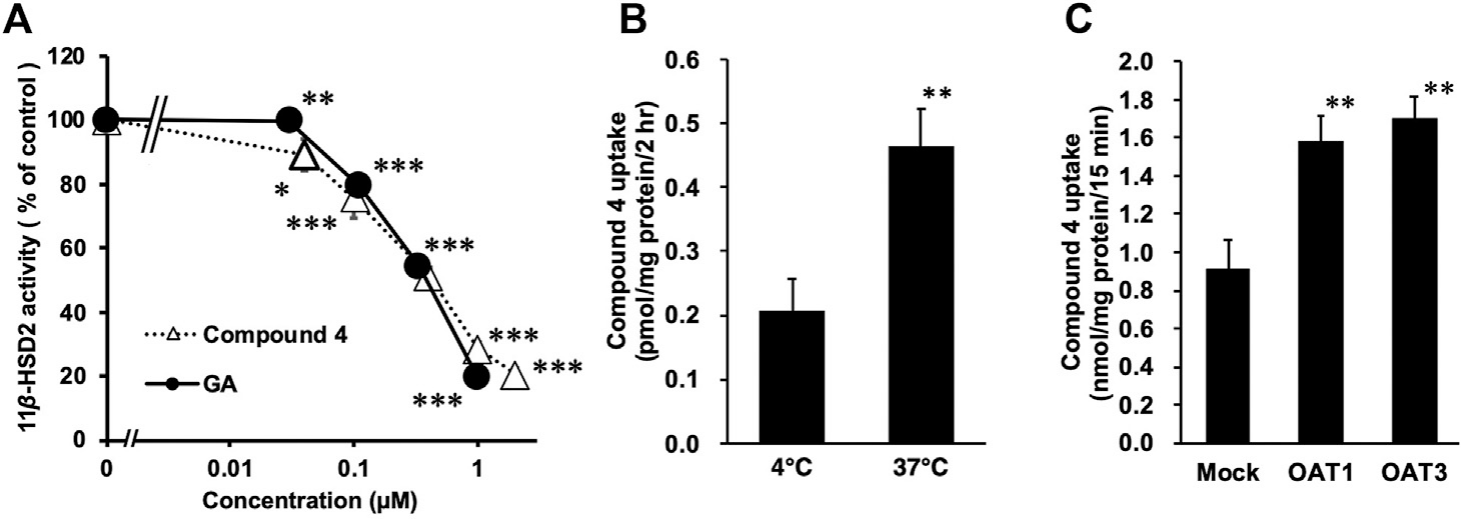
Inhibitory effects of compound **4** on type-2 11*β*-hydroxysteroid dehydrogenase (11*β*-HSD2) using rat kidney microsome (A), and the uptake of **4** into rat kidney slices (B) or into HEK293 cells transfected with organic anion transporter (OAT) 1 (C) or OAT3 (D). **(A)** [^3^H] cortisone and glycyrrhetinic acid (GA) or **4** were mixed with rat kidney microsome fractions, and incubated at 37°C for 30 min. Then, the amount of [^3^H] cortisol was measured. Data are means ± standard error (S.E: *n* = 4) of percentage [^3^H] cortisol in mixtures without samples. *p < 0.05, **p < 0.01 and ***p < 0.001 compared with groups without samples using Dunnett’s multiple *t*-test **(B)** Female Eisai hyperbilirubinuria rats (EHBRs) were orally treated with GA (200 mg/kg), and their plasma samples were collected 24 h after the treatment. Concentration of **4** in plasma sample was 115 µM. Kidneys were collected from normal Sprague-Dawley (SD) rats and slices were incubated with plasma samples of female EHBRs at 37°C or 4°C for 2 h, and then homogenized. Samples were those described in our previous study (Ishiuchi et al., 2019). Concentrations of **4** in kidney slice homogenates were measured using liquid chromatography-tandem mass spectrometry (LC-MS/MS). **p < 0.01 *vs* 4°C group using Student’s *t*-test **(C)** Male EHBRs were orally treated with GA (200 mg/kg) and their plasma samples were collected 24 h after treatment. Concentrations of **4** in plasma sample was 189 μM. HEK293 cells transfected with OAT1 or OAT3 were incubated with 1:2 diluted plasma samples of male EHBRs at 37°C for 15 min. Then, concentrations of **4** in cells were measured using LC-MS/MS. Data are means ± S.E. (*n* = 6). *p < 0.05 *vs* mock cells using Dunnett’s multiple *t*-test.

**FIGURE 3:**
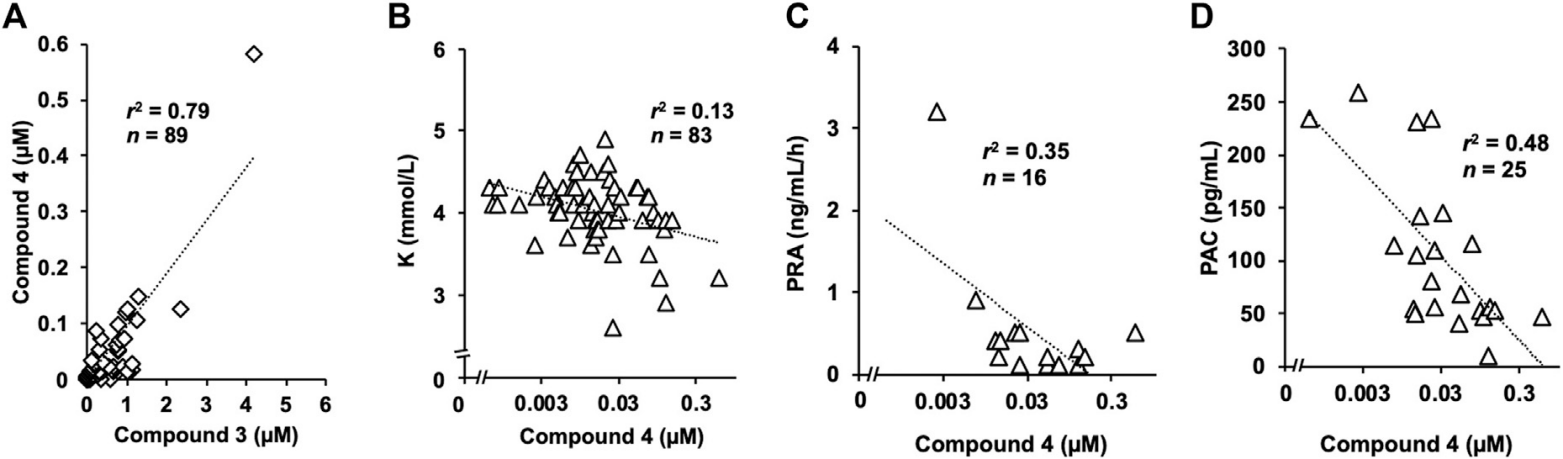
Concentration of 4 in human serum samples and three or laboratory markers **(A)** Strong positive correlation was identified between serum concentrations of **3** and **4**. The concentration of **4** was approximately 10-fold lower than that of **3 (B)** Serum potassium concentration tended to be lower in participants with higher serum concentration of compound **4 (C)** Plasma renin activity and **(D)** plasma aldosterone concentration were also suppressed in patients with higher concentration of compound **4**, which were negatively correlated.

**FIGURE 4:**
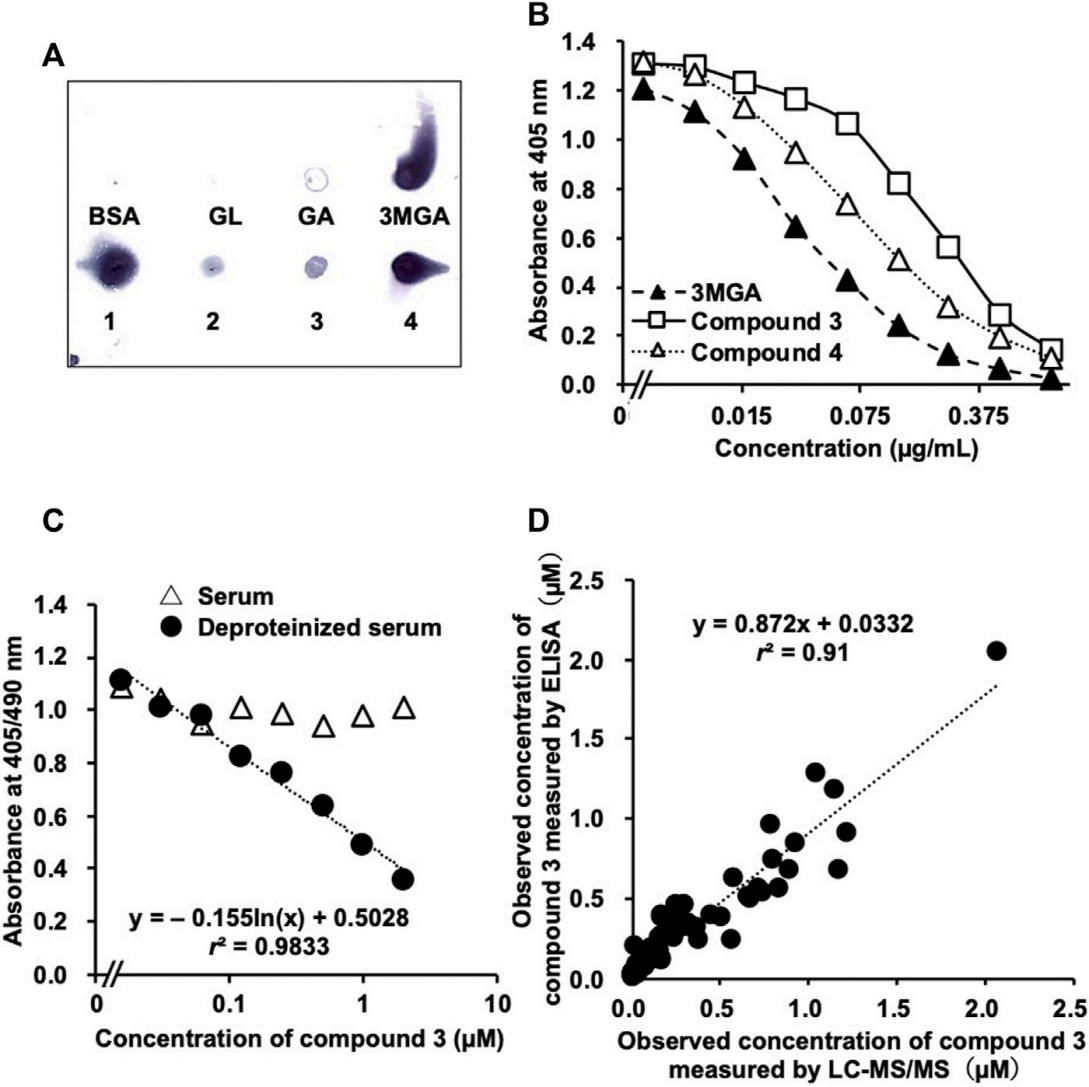
Development of enzyme-linked immunosorbent assay (ELISA) system to measure concentrations of **3** in human serum samples using anti-18*β*-glycyrrhetyl-3-*O*-glucuronide (3MGA)-monoclonal antibody (mAb). **(A)** Dot-blot analysis of glycyrrhizin (GL) metabolites using anti-3MGA-mAb. GL metabolites and bovine serum albumin (BSA, 1 µg each) were spotted onto polyethersulfone membrane and stained using an anti-3MGA-mAb. **(B)** Competitive ELISA using an anti-3MGA-mAb for 3MGA, **3**, and **4** in aqueous solution was performed **(C)** Standard solutions of **3** (15.6 nM–2 µM) in normal human serum were prepared. Serum samples were deproteinized by treating with 80% ethanol, followed by centrifugation, and the supernatants were dried up under reduced pressure. Standard lines between absorbance and concentrations of compound **3** are shown **(D)** Accuracy of ELISA system in measuring concentrations of **3** in human serum samples using anti-3MGA-mAb. Human serum samples were deproteinized, and concentration of **3** was measured using ELISA system. Samples with observed values higher than 400 nM were diluted appropriately with H_2_O to set detectable range from 10 to 400 nM. Values were compared with the result of liquid chromatography-tandem mass spectrometry (LC-MS/MS) analysis from our previous study (Takahashi K, 2019). Significant relationship was observed between the values observed using ELISA and LC-MS/MS values (*r* ^2^ = 0.91).

Statistical analysis of the inhibitory effect of **4** on 11*β-*HSD2 (Figure 2) and the results of the uptake study using OAT1-and OAT3-induced cells (Figure 2C) were performed using a one-way analysis of variance (ANOVA), followed by Dunnett’s multiple *t*-test using PASW Statistics (version 18, SPSS; IBM, Armonk, NY, United States). Statistical analysis of the uptake study using rat kidney slices (Figure 2B) was performed using the Student’s *t*-test with the Microsoft Excel^®^. The calibrated line and the regression formula in Figures 3, 4D were calculated by the linear least-squares method along with Pearson’s product moment correlation coefficients using the Microsoft Excel^®^. A probability value of less than 0.05 was considered statistically significant.

## Results

### Isolation and Structural Elucidation of Compound 4 From EHBR Urine

From the urine of female EHBRs, we isolated compound **4** (3.2 mg) along with **2** and **3**. Compound **4** [[α]_D_^24^ + 107 (*c* 0.3, methanol)] exhibited a deprotonated molecule at *m/z* 725 (M-H)^-^ in the ESIMS, and the molecular formula, C_36_H_54_O_13_S, was established by HRESIMS [*m/z* 725.3212 (M-H)^-^, *Δ*+0.5 mmu]. The ^1^H and ^13^C NMR (Table 1) and heteronuclear single quantum coherence (HSQC) spectra of **4** shared similarity with those of 22*α*-hydroxy-18*β*-glycyrrhetyl-3-*O*-sulfate-30-glucuronide (**1**) except for five signals (*δ*_C_ 38.7, *δ*_H_ 1.45 and 1.38; *δ*_C_ 32.9; *δ*_C_ 32.0, *δ*_H_ 2.01 and 1.45; *δ*_C_ 28.9, *δ*_H_ 0.82; and *δ*_C_ 27.4, *δ*_H_ 2.15, and 1.03) in **4**. In particular, compound **4** shows the disappearance of one methine signal (*δ*_C_ 76.1, *δ*_H_ 3.42) at the C-22 of **1** and the difference in the molecular formulas between **4** and **1** indicated that compound **4** was a deoxygenated derivative of **1**.

The planar structure of **4** was elucidated by analysing the two-dimensional (2D) NMR data including the ^1^H-^1^H correlation spectroscopy (COSY), HSQC, and heteronuclear multiple bond correlation (HMBC) spectra in CD_3_OD. The analysis ^1^H-^1^H COSY spectrum disclosed six structural units (C-1−C-3, C-5−C-7, C-15−C-16, C-18−C-19, C-21−C-22, and C-1′−C-5′). The observed HMBC correlations including a key HMBC cross-peaks of H_3_-28 (*δ*_H_ 0.82) to C-16 (*δ*_C_ 27.4), C-17 (*δ*_C_ 32.9), C-18 (*δ*_C_ 49.3), and C-22 (*δ*_C_ 38.7) revealed that the planar structure of **4** was the same as that of **1** deoxygenated at C-22 (Table 1 and Figure 1B). The connections of a *β*-glucuronic acid group to C-30 and of a sulfate group to C-3 were suggested by the HMBC cross-peaks of H-1′ (*δ*_H_ 5.53) to C-30 (*δ*_C_ 176.9) and H_3_-24 (*δ*_H_ 0.86) to C-3 (*δ*_C_ 87.3), respectively (Figure 1B). The relative stereochemistry of **4** was revealed to be the same as that of **1**, except for the C-22 based on the rotating frame overhauser effect spectroscopy (ROESY) correlations for H-3/H-5, H_3_-24/H_3_-25, H-9/H-5, and H_3_-27; H-6b/H_3_-25 and H_3_-26; H_3_-28/H-15a, H-16b, and H-18; H_3_-29/H-19a and H-19b; H-16b/H-22b, H-18/H-22a, and H-1′/H-3′; and H-5′ and based on 3J_H-1′/H-2′_ (8.0 Hz) and 3J_H-4′/H-5′_ (9.0 Hz, Figure 1C). The ECD spectrum of **4** showed a positive Cotton effect at 230 nm, similar to that of **1**. Thus, the absolute configuration of **4** was established as 3*S*, 5*R*, 8*R*, 9*R*, 10*S*, 17*R*, 18*S*, and 20*R*. A metabolite possessing the same structure as that of **4** was speculated in rat bile after intravenous injection of GA in a previous LC-MS analysis by Jing et al. (2008). However, to the best of our knowledge, we successfully purified compound **4** and elucidated the structure comprehensively for the first time in this study.

### Inhibitory Effects of Compound 4 on Rat 11*β*-HSD2

Compound **4** and GA (positive control) significantly inhibited 11*β*-HSD2 in the rat kidney microsome fraction in a concentration-dependent manner (Figure 2A) with half-maximum inhibitory concentration (IC_50_) values of 0.38 and 0.35 µM, respectively.

### Transport of Compound 4 Into Rat Kidney Slices and Cells Expressing OAT1 and 3

The pooled plasma samples collected from EHBRs 12 h after oral treatment with GA were loaded into ultracentrifuge filters with a 1 × 10^4^ molecular weight cut-off and concentrated by centrifugation. The concentrations of **4** in the pooled plasma and the filtrate were 77 µM and undetectable, respectively. Based on the detection limits (3.2 nM) in the LC-MS/MS analysis, the albumin-binding ratios of **4** in the plasma was calculated to be more than 99.9%.

Kidney slices prepared from Sprague-Dawley (SD) rats were incubated with the pooled plasma at 4°C or 37°C for 2 h. Figure 2B shows the amounts of **4** accumulated in the kidney slices. The uptake of **4** by the kidney slices was significantly higher at 37°C (*p* < 0.01) than at 4°C (Figure 2B).

HEK293 cells transfected with OAT1, OAT3, or the mock plasmid were incubated with pooled plasma collected from EHBRs at 37°C for 15 min. The uptake of **4** into the cells was then measured (Figure 2C) and its level in cells expressing OAT1 or OAT3 was significantly higher (*p* < 0.01) than in mock cells.

### Detection of Compound 4 in Human Serum Samples

Among the 89 serum samples stored, the daily dose of liquorice was available for samples from 86 participants, and 70 participants were taking liquorice-containing herbal preparations when we collected the serum samples. We detected compound **4** in 62 samples from the 70 participants. In contrast, we detected trace amounts of compound **4** in only one sample from the 16 participants who were not taking liquorice-containing herbal preparations. However, we found a weak positive correlation between the serum concentrations of compound **4** and the daily dosage of liquorice (Supplementary Figure S1A). On the other hand, we found a strong positive correlation between serum concentrations of **3** and **4**, and the concentration of **4** was approximately 10-fold lower than that of **3** (Figure 3A). We detected trace levels of compound **4** (3 nM) in one urine sample among the 20 available samples.

### Relationship Between Laboratory Markers or Clinical Symptoms of Pseudohyperaldosteronism and Serum Concentration of Compound 4

Serum potassium concentration tended to be lower in participants with higher serum concentrations of compound **4** (Figure 3B). We confirmed a pseudohyperaldosteronism case where the patient had normal potassium concentration. The serum sample was collected from the patient who had been administered spironolactone which could increase potassium concentration. Other patients with higher serum concentrations of compound **4** had not been administered potassium-sparing medications. Additionally, there were negative correlations between the serum concentration of **4** and both plasma renin activity and aldosterone concentration (Figures 3C,D activated renin activity is shown in Supplementary Figure S1B). The relationship between compound **4** and clinical symptoms was not significant. The systolic and diastolic blood pressure were not measured on the day we collected the serum samples (Supplementary Figure S1C) and exacerbation of hypertension (data not shown) was not correlated with the concentration of compound **4**. No relationship was found between compound **4** and oedema grade (Supplementary Figure S1D) and exacerbation (data not shown).

### Dot-Blot Analysis and the Cross-Reactivity of Compound 4 to anti-3MGA-mAb

We confirmed the cross-reactivity of anti-3MGA-mAb with GL, GA, 3MGA, and compounds **1**–**4** dissolved in aqueous solution. The anti-3MGA-mAb cross-reacted with **4** at a level similar to that of **1**, followed by **3** and **2** (Figure 4A). The concentration profiles of **3** and **4** to the absorbance in a competitive ELISA system using anti-3MGA-mAb were well characterised, and when the specificity of anti-3MGA-mAb to 3MGA was calibrated to 100%, the cross-reactivity for **3** and **4** was 23 and 40%, respectively (Figure 4B).

### Measurement of the Concentration of Compound 3 Using ELISA With anti-3MGA-mAb

When **3** was dissolved in normal human serum and its concentration was measured using ELISA, no competitive reaction was observed between **3** and anti-3-MGA-mAb, and the standard line between the concentration of **3** and the absorbance could not be plotted. After deproteinization using 80% ethanol, a better standard line was obtained (Figure 4C). Using this protocol, we measured the concentration of **3** in 97 human serum samples and compared the data with those measured using the LC-MS/MS method described in our previous study (Takahashi et al., 2019). However, the *r* ^2^ value between the obtained data measured using ELISA and LC-MS/MS was 0.69, and inconsistencies appeared at higher concentrations. When the first measurements were more than 400 nM, the serum samples were diluted 5- or 10-fold with H_2_O, and the concentration of **3** was measured again using the same ELISA system. This produced a better correlation (*r* ^2^ = 0.91) between the data measured using ELISA and LC-MS/MS (Figure 4D).

### Pharmacokinetics of GA Metabolites in Female and Male EHBRs Orally Treated With GA

**FIGURE 5:**
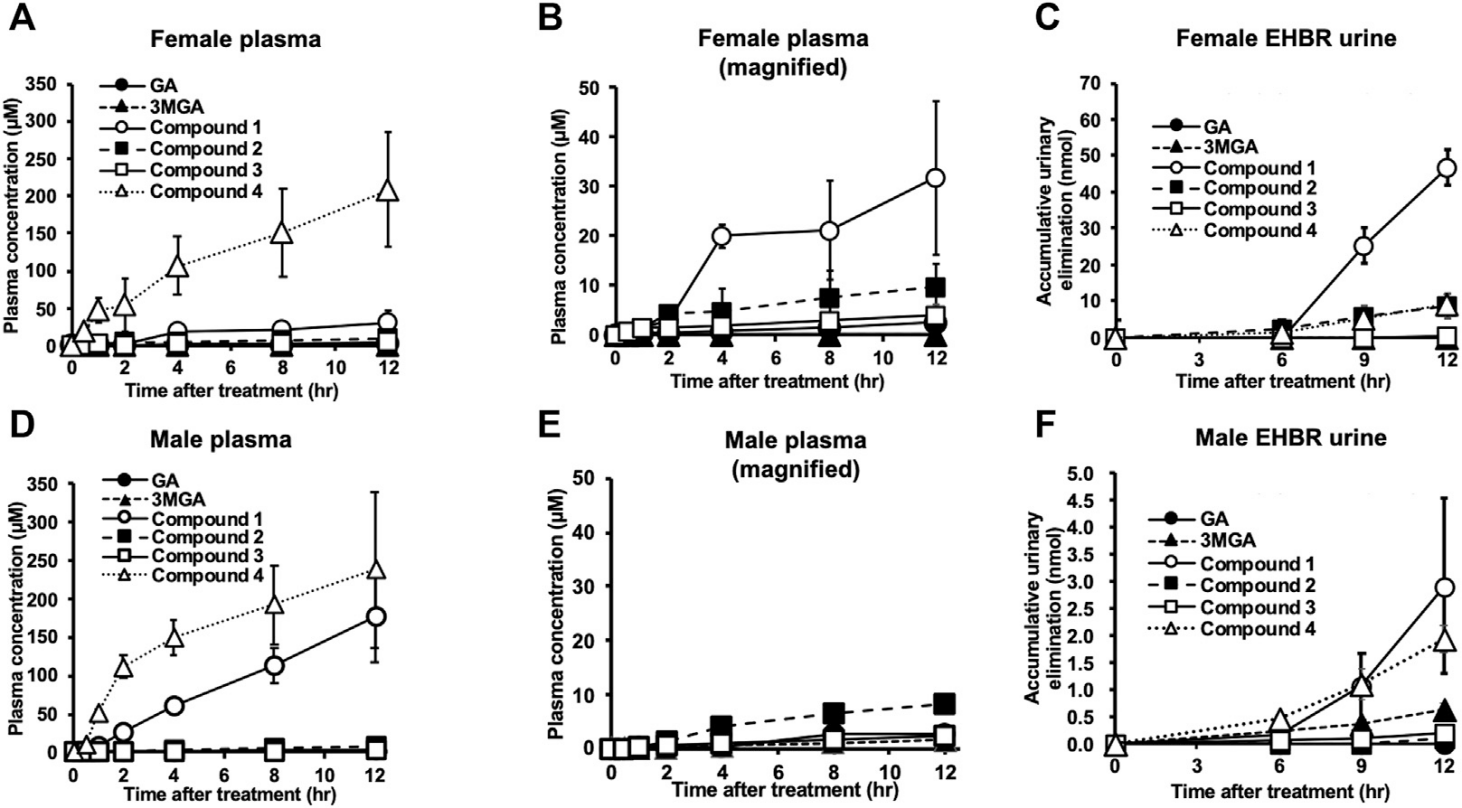
Pharmacokinetic profiles of glycyrrhizin (GL) metabolites in female **(A–C)** and male **(D–F)** Eisai hyperbilirubinuria rats (EHBRs). Glycyrrhetinic acid (GA, 200 mg/kg) was administered orally to anesthetized EHBRs, and plasma and urine were collected for 12 h. Data of GA, 3MGA, **1**, **2**, and **3** in **(A)** and GA, 3MGA, **2**, and **3** for **(D)** are relatively small; their magnified graphs are shown in **(B,E)**, respectively. Concentrations of GA metabolites were measured using liquid chromatography-tandem mass spectrometry (LC-MS/MS), and data are means ± standard error (S.E: *n* = 4).

We successively collected plasma and urine samples from both male and female EHBRs that were orally administered GA (0.20 g/kg), and measured the concentrations of GA and its metabolites using LC-MS/MS. Figure 5 shows the plasma concentration profiles and urinary elimination of GA and its metabolites. In female EHBRs orally treated with GA, **4** appeared in the plasma after 30 min and the concentration of **4** in plasma was sequentially increased over 12 h (Figure 5A). Next, the concentrations of **1**, **2**, and **3** were gradually increased after the oral treatment with GA (Figure 5B). The concentrations of GA and its metabolites 12 h after oral administration of GA in female EHBRs were 2.7 µM of GA, 0.1 µM of 3MGA, 32 µM of **1**, 10 µM of **2**, 2.0 µM of **3**, and 208 µM of **4**. The urine of female EHBRs showed the highest elimination levels of **1**, followed by **2** and **4**, which were at the same levels (Figure 5C). In male EHBRs, although the profiles of the plasma concentrations of **4**, **2**, and **3** exhibited curved similar to those of female EHBRs, the concentration curve of **1** was much higher than that of the female EHBRs (Figures 5D,E). The concentrations of GA and its metabolites 12 h after oral administration of GA in male EHBRs were 2.6 µM of GA, 1.6 µM of 3MGA, 177 µM of **1**, 8.2 µM of **2**, 2.2 µM of **3**, and 237 µM of **4**. In the urine of male EHBRs, the elimination of these metabolites was approximately 10-fold lower than it was in female EHBRs (Figure 5F). The eliminations of **1** and **4** were the highest and at similar levels, respectively. The plasma concentration and urinary elimination of 3MGA in male EHBRs was tended to be higher than those in female EHBRs.

**FIGURE 6:**
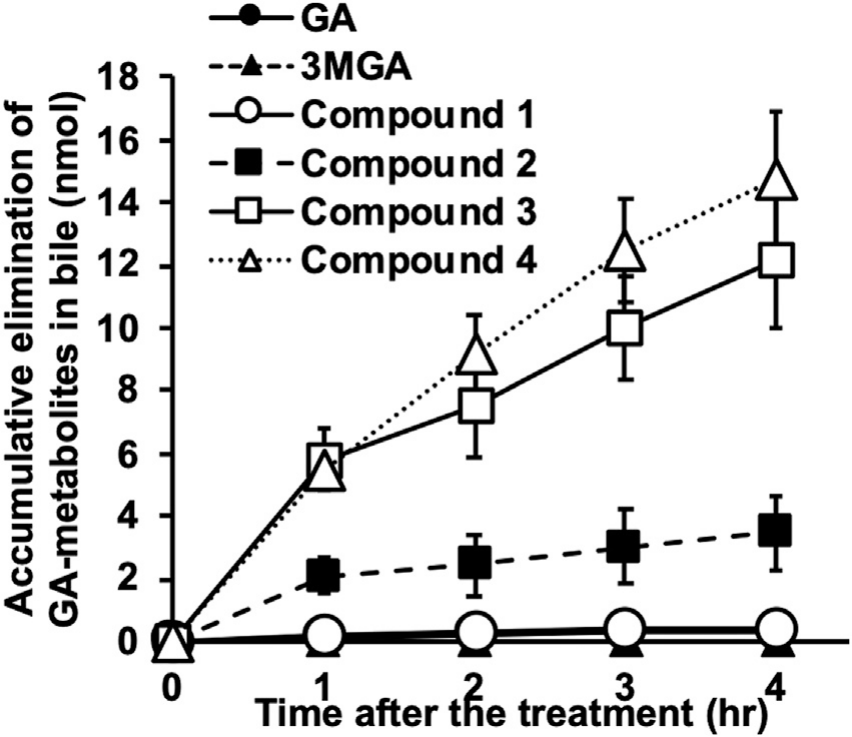
Elimination of glycyrrhizin (GL) metabolites into bile in Sprague-Dawley (SD) rats. Female SD rats (10 week-old) were anesthetized using intraperitoneal injection of urethane (1 g/kg), and biliary tracts were cannulated. Glycyrrhetinic acid (GA, 0.2 mg/kg) was injected into jugular veins and bile was collected every hour for 4 h. Concentrations of GA metabolites were measured using liquid chromatography-tandem mass spectrometry (LC-MS/MS) and accumulated elimination of GA-metabolites are means ± standard error (S.E: *n* = 3).

These metabolites were gradually excreted into the bile in female SD rats with functional Mrp2 which were intravenously injected with GA, and the accumulation of **4** in the bile was the highest, followed by **3** and **2** (Figure 6). The accumulative elimination of **2**, **3**, and GA in faeces collected 24 h after the injection were 0.86 ± 0.1 nmol, 1.8 ± 0.3 nmol, and 9.8 ± 1.3 nmol, respectively. however, GL, 3MGA, **1** and **4** in faeces were below detectable levels. Compounds **1**–**4** were not detected in the serum of female SD rats collected 1, 2, 3, 4, and 24 h after GA injection.

## Discussion

We found compounds **1**, **2**, and **3** in the urine of EHBR orally treated with GA and proposed that these compounds could be candidates agents, along with the previously identified 3MGA (Kato et al., 1995; Makino et al., 2008; Makino et al., 2012), causing GL-induced pseudohyperaldosteronism (Morinaga et al., 2018; Ishiuchi et al., 2019). We analyzed 97 serum samples from patients suspected to have developed pseudohyperaldosteronism and found that **3** was the most probable causative agent (Takahashi et al., 2019). In this study, we isolated **4** as a new GL metabolite from the urine of EHBR orally treated with GA. Compound **4** exhibited 11*β*-HSD2 inhibitory activity equivalent to that of GA and high binding activity to serum albumin; additionally compound **4** was recognized as a substrate for OAT1 and OAT3 to transfer into renal tubular epithelial cells where 11*β*-HSD2 is expressed. When we measured the concentration of **4** in human serum samples from patients who developed pseudohyperaldosteronism, the concentration detected was sufficient but lower than that of **3**. This observation suggests that **4** is also a candidate causative agent of GL-induced pseudohyperaldosteronism.

Our results suggest that **3** is the major GL metabolite in human serum, followed by **4**, which can reach 11*β*-HSD2 in renal tubular epithelial cells. The analysis of serum samples from most of patients indicated that those with pseudohyperaldosteronism did not contain **1** or **2**, and the major GL metabolites in blood of these patients was **3** which was found at 1.5–2-fold higher than those of GA, and there was a positive correlation between the blood concentrations of **3** and GA (Takahashi et al., 2019). In the present study, **4** was detected in the same serum samples at concentrations of approximately 10-fold lower than those of **3**, and their concentrations were positively correlated. The serum concentration of **4** was negatively correlated with several blood markers of pseudohyperaldosteronism such as serum potassium concentration, plasma renin activity, and plasma aldosterone concentration. The serum concentration of **4** was lower than that of **3** in the patients and the inhibitory effect of **4** on 11*β*-HSD2 was also lower than that of **3** as the IC_50_ values of **3** and **4** on 11*β*-HSD2 were 0.10 µM (Ishiuchi et al., 2019) and 0.38 µM, respectively. Therefore, **3** would be more effective than **4** and could cause pseudohyperaldosteronism in patients using liquorice or GL-preparations.

To prevent the development of pseudohyperaldosteronism, early laboratory tests to detect the blood concentration of **3** in patients prescribed with liquorice-containing medicines are desirable. The analysis of **3** using LC-MS/MS has the disadvantage of requiring elaborate facilities and is not common in general hospitals, clinics, and pharmacies because of its high cost and long processing time. These disadvantages could be overcome with the ELISA system which is more convenient with a lower price and faster analysis in the detection of specific compounds in biological samples. In our previous study, we proposed that the blood concentration of **3** in the patients orally treated with liquorice could be measured using ELISA to prevent the onset of pseudohyperaldosteronism in early stages by using an anti-3MGA-mAb because of the enough affinity of anti-3MGA-mAb to **3** (Ishiuchi et al., 2019). However, we could not measure the concentration of **3** in the serum samples of the patients by using this ELISA system. Because major portions of **1–4** in serum are bound to albumin, the recognition site of this antibody to **3** has been predicted to likely the same as the binding site of **3** to albumin. Treating the serum samples with ethanol and deproteinizing them enabled the construction of a good calibration curve by using the standard human serum solution of **3** and the absorbance obtained using ELISA. Then, the concentrations of **3** in the serum samples of the patients were measured using ELISA after the serum samples were treated with ethanol. The comparative analysis of the methods showed higher values were detected using ELISA than LC-MS/MS, especially at high concentrations of **3**, and many outliers were observed in the correlation. The affinity of anti-3MGA-mAb to **4** was higher than to **3**, suggesting that the cross-reaction of anti-3MGA-mAb to **4**, as to the minor metabolite in the serum, could not be negligible in the samples with high concentration of **3**. To avoid the cross-reaction of this antibody to **4**, the measurable range of **3** in ELISA should be set to 10–400 nM. When compound **3** was obtained at more than 400 nM in the first measurement, the serum samples were appropriately diluted and re-measured. This process produced a good correlation of the values obtained using ELISA with those using LC-MS/MS. Furthermore, metabolites other than **3** and **4** that could cross-react with this antibody were considered likely not present in human serum. Then, the ELISA measurement system for the serum concentration of **3** was established and it is expected to contribute to the prevention of pseudohyperaldosteronism in patients administered liquorice-containing medicines.

In female SD rats, GA was excreted mainly in the bile as **4** and **3**, and as the minor metabolites **2** and **1** when GA was administered intravenously. These metabolites did not appear in the blood of these rats, whereas they appeared in that of EHBRs. EHBRs were originally found as mutant rats with chronic conjugated hyperbilirubinemia (Hosokawa et al., 1992). They were subsequently confirmed to express the dysfunctional Mrp2 protein by a point mutation in the open reading frame (Ito et al., 1997). Compounds **1** – **4** did not appear in the blood and urine of SD rats but in those of EHBRs, it is suggested that Mrp2 was involved in the bile excretion of these metabolites, and the reduction and/or dysfunction of Mrp2 by liver injury or mutation decreased bile excretion of these metabolites. In normal SD rats, GA can be sulfonated by Sult2A1 (Takahashi et al., 2019) to generate **3**, or sulfonated and glucuronidated to generate **4** in the liver, and then **3** and **4** can be excreted into the bile *via* Mrp2. In the gastrointestinal tract, **3** and **4** can be hydrolysed by intestinal bacteria to GA, which can then be partly absorbed from the gastrointestinal tract into the circulation, while the other part is excreted into faeces. Indeed, we found **3** and GA in the faeces of SD rats injected intravenously with GA (Ishiuchi et al., 2019). Although **4** was more highly excreted into the bile than **3**, it was not detected in faeces, suggesting that the hydrolysis reaction mediated by enterobacteria at C-30 was superior to that at the C-3 of **3** and **4**.

In EHBRs, compounds **1**–**4** appeared in the blood as GL metabolites, but there was a difference in their blood concentrations between male and female EHBRs. The blood concentration of **1** in male rats was approximately 7-fold higher than that in female rats 12 h after oral administration of GA, indicating a marked sex difference. We then evaluated the possibility of sex differences in blood concentrations of **3** and **4** among the patients, but none was observed (data not shown). The possible mechanism of the sex difference observed in EHBRs may be related to the hydroxylating function of Cyps on GA-metabolites at C-22. The observation that the serum samples collected from patients did not contain **1** or **2** (Takahashi et al., 2019) indicated that the activity of CYPs involved in the hydroxylation of GA and its metabolites at C-22 could be much lower in human beings than that in rats, and, therefore, it was reasonable that we could not find sex differences in blood concentrations of **3** and **4** in serum samples of patients.

Although the functions of MRP2 were not directly evaluated in the present patients, their functions were not considered not to have varied significantly. This is because serum concentrations of direct bilirubin, as a potential surrogate marker of MRP2 function, were normal in all the participants in this study. Furthermore, there was a positive correlation between the daily dose of liquorice and blood concentrations of **3** and **4**. We detected very small amounts of **3** (Ishiuchi et al., 2019) and **4** (data not shown) in plasma samples collected from normal SD rats orally treated with GA. It is suggested that the functions of human MRP2 and rat Mrp2 may be different, and human MRP2 likely transports **3** and **4** less efficiently than rat Mrp2 does.

Previous studies suggested GA as the causative agent of pseudohyperaldosteronism (Ulmann et al., 1975; Armanini et al., 1983; Monder et al., 1989) with the binding activity (ED_50_) of GA on mineralocorticoid receptor (MR) being about 100 µM. In our previous clinical study, the maximum concentrations of compound **3** and GA are 4.2 µM and 1.8 µM, respectively (Takahashi et al., 2019). The serum concentration of GA was too low not to inhibit MR directly. Although the affinity of compounds **3** and **4** for MR is unknown and the possible future studies are needed, it is estimated that the affinities would be low by the previous results of GA (Armanini et al., 1983). Indeed, there are several earlier reports (Molhuysen et al., 1950; Borst et al., 1953; Card et al., 1953; Elmadjian et al., 1956; Girerd et al., 1960) exhibiting that the adrenal gland must be present for liquorice to have mineralocorticoid activity, suggesting that glycyrrhizin and its metabolites itself including GA do not bind to MR directly *in vivo*. We have also addressed the capability of GA on transporting through the membrane of tubular cells, and shown that GA could not transfer into the cells under the existence of albumin (Makino et al., 2012). In the study using rat kidney slices, GA can only bind the surface of the tissue, and cannot exhibit any binding effect of mineralocorticoid receptor and the inhibition of 11*β*-HSD2 at 100 µM (Makino et al., 2012). Therefore, we suggest that GA might not be the causative agent of pseudohyperaldosteronism induced by liquorice *in vivo*.

The limitation of this study is the possibility that there are other GL metabolites than compounds **3** and **4** in the serum of patients who took liquorice. Ploeger et al. predicted 18*β*-glycyrrhetyl-30-*O*-glucuronide as another metabolite of GL than the metabolites evaluated in this study (Ploeger et al., 2001). However, the possibility of its presence may be low because we found no peaks in MS chromatogram of ESI(+) 647.6 to 453.6 m*/z* for 3MGA in all human samples. In MS chromatogram of ESI(+) 471.3 to 91.0 m*/z* for GA, we found other possible GL metabolites than compounds **3** and **4**, though the size of these peaks are quite smaller than **3** and **4** (data not shown). In order to reveal the causative agents of liquorice-induced pseudohyperaldosteronism completely, metabolomics studies will be demanded.

Collectively, our results led us conclude that compound **3** would be the most promising causative agent of pseudohyperaldosteronism. Furthermore, **3** was detected in the human serum at concentrations 1.5- to 2-fold higher than those of GA, implying that **3** also acted as the active ingredient exerting not only pseudohyperaldosteronism but also other pharmacological actions such as the anti-inflammatory effects of GL and liquorice preparations (Isbrucker and Burdock, 2006) in human beings. Further clinical studies are needed to investigate the pharmacologically and toxicologically active ingredients of GL and liquorice.

## Conclusion

We identified **3** as the major metabolite and **4** as the secondary major GL metabolite, in the blood of patients administered GL and liquorice, which could reach 11*β-*HSD2 in renal tubular epithelial cells. We concluded that **3** was the most promising causative agent of pseudohyperaldosteronism induced by liquorice, and we successfully established an ELISA system to easily detect the blood concentration of **3** in patients taking liquorice, to prevent the onset of pseudohyperaldosteronism. Further pharmacological studies of **3** are required to determine the mechanisms of action of GL and liquorice preparations in humans.

## Data Availability

The raw data supporting the conclusions of this article will be made available by the authors, without undue reservation.
