# Corrigendum: Identification of an alternative glycyrrhizin metabolite causing liquorice-induced pseudohyperaldosteronism and the development of ELISA system to detect the predictive biomarker

**DOI:** 10.3389/fphar.2022.1090327

**Published:** 2022-11-24

**Authors:** Kan’ichiro Ishiuchi, Osamu Morinaga, Tetsuhiro Yoshino, Miaki Mitamura, Asuka Hirasawa, Yasuhito Maki, Yuuna Tashita, Tsubasa Kondo, Kakuyou Ogawa, Fangyi Lian, Keiko Ogawa-Ochiai, Kiyoshi Minamizawa, Takao Namiki, Masaru Mimura, Kenji Watanabe, Toshiaki Makino

**Affiliations:** ^1^ Department of Pharmacognosy, Graduate School of Pharmaceutical Sciences, Nagoya City University, Nagoya, Japan; ^2^ Department of Natural Medicines, Daiichi University of Pharmacy, Fukuoka, Japan; ^3^ Center for Kampo Medicine, Keio University School of Medicine, Tokyo, Japan; ^4^ Department of Otorhinolaryngology and Head and Neck Surgery, Clinic of Japanese Oriental (Kampo) Medicine, Kanazawa University Hospital, Kanazawa, Japan; ^5^ Department of Oriental Medicine, Kameda Medical Center, Kamogawa, Japan; ^6^ Department of Japanese Oriental (Kampo) Medicine, Graduate School of Medicine, Chiba University, Chiba, Japan

**Keywords:** kampo medicine, side effect, liquorice, glycyrrhizin, pseudoaldosteronism, sex differences

In the published article, there was an error. The numbers of the concentrations were mistaken.

A correction has been made to **Results**, *Pharmacokinetics of GA metabolites in female and male EHBRs orally treated with GA*, Paragraph 7. These sentences previously stated:

“The concentrations of GA and its metabolites 12 h after oral administration of GA in female EHBRs were 3.2 µM of GA, 0.1 µM of 3MGA, 14 µM of **1**, 4.3 µM of **2**, 6.6 µM of **3**, and 166 µM of **4**.”

and

“The concentrations of GA and its metabolites 12 h after oral administration of GA in male EHBRs were 2.6 µM of GA, 1.2 µM of 3MGA, 102 µM of **1**, 4.1 µM of **2**, 1.2 µM of **3**, and 198 µM of **4**.”

The corrected sentences appear below:

“The concentrations of GA and its metabolites 12 h after oral administration of GA in female EHBRs were 2.7 µM of GA, 0.1 µM of 3MGA, 32 µM of **1**, 10 µM of **2**, 2.0 µM of **3**, and 208 µM of **4**.”

and

“The concentrations of GA and its metabolites 12 h after oral administration of GA in male EHBRs were 2.6 µM of GA, 1.6 µM of 3MGA, 177 µM of **1**, 8.2 µM of **2**, 2.2 µM of **3**, and 237 µM of **4**.”

The authors apologize for this error and state that this does not change the scientific conclusions of the article in any way. The original article has been updated.

