# Supplementary material for: Identification of an Alternative Glycyrrhizin Metabolite Causing Liquorice-Induced Pseudohyperaldosteronism and the Development of ELISA System to Detect the Predictive Biomarker
Source: Front Pharmacol. 2021 May 17;12:688508. doi: 10.3389/fphar.2021.688508 (PMC8165744; doi:10.3389/fphar.2021.688508)
Supplement: Supplementary file 1 [file DataSheet1.PDF]

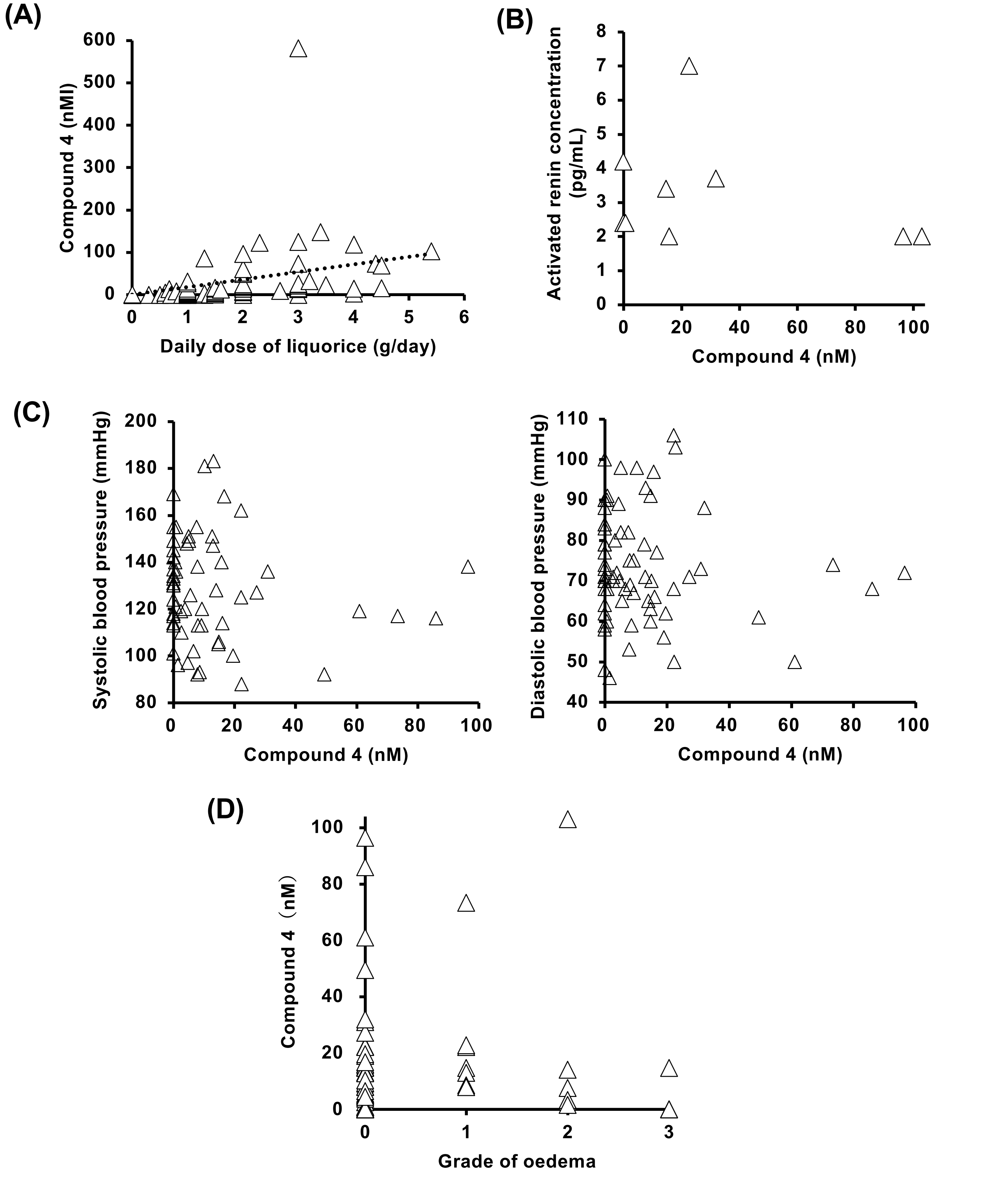

Supplementary Fig. 1 (A) Serum concentration of compound **4** and daily liquorice dose. Weak correlation between serum concentration of compound **4** and daily liquorice dose was found. (B) Serum concentration of compound **4** and activated renin concentration. Higher serum concentration of compound **4** tended to lower serum potassium, renin and aldosterone. (C) Serum concentration of compound **4** and blood pressure. Systolic and diastolic blood pressure on day serum samples were collected were not correlated with concentration of compound **4**. (D) Serum concentration of compound **4** and oedema. No relationship was identified between exacerbation of oedema and compound **4**. Number of horizontal axis represents grade of oedema.
